# Silent Strength: The Social Support of Mental Health Nurses

**DOI:** 10.1111/inm.70132

**Published:** 2025-09-04

**Authors:** Sinead Barry, Katrin Leifels, Ruby Walter, Azizur Rahman

**Affiliations:** ^1^ RMIT University Melbourne Victoria Australia

**Keywords:** informal support, mental health nursing, social support, stress management, well‐being

## Abstract

Mental health nurses (MHNs) face unique occupational challenges, including high emotional demands, frequent exposure to workplace violence, and risk of burnout. Social support is widely recognised as a protective factor that can enhance well‐being and job satisfaction in this high‐stress profession. In this research, social support is defined as the emotional, informational, and instrumental assistance that MHNs receive from interpersonal relationships in the workplace, such as from colleagues, immediate supervisors, and informal peer networks. HR support or organisational support, by contrast, refers to formal structures and resources provided by the organisation itself. Despite this, little is known about MHNs' perceptions of the availability, accessibility, and value of social support within their workplace settings. This qualitative study explored the perceived levels and effects of social support among MHNs using semi‐structured interviews. Six MHNs were recruited via purposive and snowball sampling. Interviews were conducted online, transcribed, and analysed thematically to identify common patterns and variations in participants' experiences. Thematic analysis identified five key themes: agency in seeking support, forming alliances, the informal nature of support, its varying availability, and the necessity of support to sustain a career in MHN. Peer support was described as essential, informal, and self‐initiated, while formal organisational support was often viewed as lacking. Digital platforms emerged as supplementary sources. The findings underscore social support's crucial role in MHNs resilience and well‐being, and highlight the need for healthcare organisations to strengthen both informal and formal support structures to improve job satisfaction, reduce burnout, and promote retention.

## 1 Introduction

Mental health nurses (MHNs) face the dual challenge of addressing clients' mental health issues whilst also maintaining their own well‐being. MHNs often experience greater demands compared to those in other professions, including the risk of violence and harassment from clients (Evans et al. 2006; Ravalier et al. 2021; Roelen et al. 2018). Research indicates that MHNs typically work in high‐pressure environments with intense workloads, and limited resources, leading to a high prevalence of exhaustion and burnout within this profession (Edwards et al. 2000; Evans et al. 2006; Hamaideh 2011; Hamama et al. 2019; Stanley and Sebastine 2023). Social support can serve as a vital job resource (Hamama et al. 2019), helping individuals manage work‐related stress and enhance overall well‐being. Defined as “information from others that one is loved and cared for, esteemed and valued, and part of a network of communication and mutual obligations” (Kim et al. 2008, 518) social support is a protective workplace factor that can support MHNs.

MHNs face unique challenges in their profession, frequently encountering high levels of stress and emotional demands that can be ameliorated by social support. Research shows that adequate support, whether emotional, instrumental, or affiliative, from colleagues and supervisors helps nurses to manage job pressures and reduces the risk of burnout (Chen et al. 2020; Hamama et al. 2019; Pergol‐Metko et al. 2023; Portela et al. 2015). The nature of mental health nursing exposes nurses to increased levels of aggression and violence, which can negatively affect their psychological well‐being (Newman et al. 2024). The effects of burnout are detrimental both physically and psychologically to an individual and directly impact the quality of consumer care, consumer health outcomes (López‐López et al. 2019), and lead to greater workforce turnover (Sharplin 2021). Therefore, in this context, social support emerges as a crucial resource to protect MHNs from workforce demands and to promote well‐being and career longevity. The benefits of social support include mitigating the effects of psychological demands on well‐being and the negative impacts of job demands (Ravalier et al. 2021; Singh et al. 2020). Previous research has confirmed well‐being and social support play a crucial role in affecting nurses' decision to remain in the profession (Hirvikallio et al. 2024; Van der Heijden et al. 2010), something the profession is grappling with due to Australia's increasing workforce shortages (Peters 2023). Social support can enhance resilience and serve as a buffer against work‐related stress for MHNs, greatly improving their overall well‐being and job satisfaction. This support can potentially decrease burnout and enhance their capacity to deliver high‐quality care to consumers.

## 2 Background

Research shows that adequate support from colleagues and supervisors helps physicians and nurses to manage job pressures and reduce the risk of burnout (Beddoe et al. 2014; Hamama et al. 2019). Social support can come from various sources, and studies indicate that the effects of support may vary depending on its source (Charoensukmongkol et al. 2016; Gariepy et al. 2016; Velando‐Soriano et al. 2020). Regular support from colleagues throughout the day can help mitigate the impact of negative interactions with clients (Carmona‐Cobo and Lopez‐Zafra 2022). Additionally, having opportunities to debrief with colleagues shortly after distressing incidents provides workers with a cathartic outlet (Singh et al. 2020). Making sense of traumatic events can also serve as a vital job resource that supports emotional processing and recovery (Hamama et al. 2019). Social support from colleagues that emerges naturally without any intervention from the organisation is categorised as informal support, whilst structured or formally provided support mechanisms, such as Employee Assistance Programs and clinical supervision, are categorised as formal support services. Support from colleagues is often more accessible than other types of support, although Fahy and Moran (2018) reported that nurses are often unaware of formal support options available and may prefer to avoid seeking support from their colleagues. Fahy and Moran (2018) also found nurses to be hesitant to approach their supervisors for emotional support regarding negative workplace experiences. Cortese et al. (2010) and Fahy and Moran (2018) suggested that managerial support may be more effective than colleague support in alleviating perceived job demands. However, evidence shows that social support from supervisors tends to decline as workers gain experience and rise in rank. While new nurses often report the benefits of emotional support and feedback from supervisors, senior nurses frequently express feelings of being unsupported by management (Gifkins et al. 2017). This may indicate an underlying assumption that experienced workers require less support at work due to their increased skills and experience.

The literature highlights the importance of job resources such as autonomy, which allows employees to make choices about their work schedules and tasks (Kim and Stoner 2008). Both social support and job autonomy interact with stress, influencing burnout and turnover intentions (Han et al. 2015; Kim and Stoner 2008; Velando‐Soriano et al. 2020). Interestingly, Gifkins et al. (2017) found that while shiftwork initially adds stress for less experienced nurses, it can become a resource that reduces burnout and attrition as nurses gain experience, control, and social support from family and friends. Currently, there is limited research on the perceptions of different types of social support and their effects across various occupations, particularly on MHNs. This research project aims to address this gap by exploring the perception and reception of social support among MHNs. Given the projected future shortfall of MHNs, it is essential to address the issues of attrition and burnout within the profession. Understanding and meeting the support needs of MHNs are crucial steps in retaining a stable and committed workforce. By focusing on these aspects, we can ensure the sustainability and effectiveness of nursing services in the future. Understanding how MHNs perceive and respond to workplace social support is essential for creating functioning formal and informal support structures.

## 3 Methods

This research project utilised a qualitative design with semi‐structured interviews conducted for data collection. Ethics approval was obtained from the University ethics committee (Approval reference 2024‐26711‐25160). The interview questions were designed based on the main research question and objectives. The study aimed to explore the perceived level of social support and its effects experienced by registered nurses working in mental health settings. Example questions included:

1. How do you perceive social support provided by your workplace?
2. How often do you receive social support?
3. How helpful do you perceive the provided level of social support?
4. Which people are the source of your support at your workplace?
5. How would you describe the adequacy of the support?
6. How would you describe the extent to which they are available when you need them?
7. How helpful do you perceive this support?

A total of 12 interviews were conducted between August and December 2023, consisting of six MHNs and six social workers working within Victoria, Australia. This paper reports on the findings of the interviews with the MHNs. The social workers' data is part of a broader project, and findings from the social workers will be reported separately. The interviews were conducted via MS Teams and recorded. Transcriptions were generated by MS Teams and reviewed by each interviewer upon completion. The interviews lasted approximately 45 to 50 min each. All recordings were deleted immediately after the checking of transcriptions was completed.

## 4 Analysis

Thematic analysis was undertaken to examine differing perspectives of research participants, including similarities and differences (Nowell et al. 2017). The thematic analysis involved a systematic approach to identifying, analysing, and reporting patterns within the qualitative data. Initially, two researchers familiarised themselves with the data through repeated readings, followed by generating initial codes to capture interesting features. These codes were then grouped into potential themes, which were reviewed and refined to ensure coherence and relevance to the research questions. Themes were defined and named, providing a clear narrative of the data. Throughout the process, reflexivity and transparency were maintained to enhance the credibility and trustworthiness of the findings. The researchers used peer debriefing as one measure to ensure the credibility of the findings, along with extended immersion in data (Lincoln and Guba 1985).

## 5 Results

The results of this study identify that social support, if available, has the potential for significant positive effect on the working experiences of MHNs. This article reports on the five key themes that emerged from mental health nurses’ experiences of social support.

### 5.1 Theme 1: Social Support Is up to You

The participant interview data revealed that the MHNs were able to readily identify social support available to them, however that the social support was largely reliant on independently developed peer connections. MHN 3 noted the importance of “*banding together and being able to support each other*”, highlighting a safety net is formed once connections are made. However, MHNs face barriers, such as limited formal opportunities for seeking social support, with MHN 1 stating “*it's very hard to practically get away and you know, unless you do it in your own time*”. MHN 4 emphasised the personal responsibility required for establishing their social support, saying, “*it's something you maintain on your own*”, giving the notion that there was no formal structure or processes in place within their organisation. Despite a “*chaotic*” work environment (MHN 5), social support is primarily self‐sought and maintained.

### 5.2 Theme 2: Social Support Is About Creating and Relying on Your Allies

This theme highlights that the MHNs primarily found social support among peers and sought this support in the moment rather than scheduling a time for a delayed reflection on an event. While some participants, like MHN 2, believed support can also come from “*senior staff and executives*”, the reality is that peer support on the ground is most accessible. Although managers may encourage social support, there are several practical barriers to accessing it. MHN 1 described, “*But it's just hard on the ground nurses who are doing a very, you know, job that requires you to be there. A lot of the time we, we're not like allied health they, you know, can sort of just block out a couple of hours, but it's harder for us*”. The majority of MHNs in this study believed that social support came from colleagues. Due to the “*chaotic…unsafe…distressing*” nature of the work (MHN 5), shared experiences with peers were seen to build strength and resilience. A further example was that peer social support stemmed from “*shared trauma*” (MHN 3) and being “*at the coal face*” together (MHN 3). These shared experiences appear to help foster a workplace culture of collegial support. MHN 3 identified that they belonged to a workplace “c*ulture that promotes people actually supporting each other at the coal face rather than necessarily having some formalised process for it*”.

### 5.3 Theme 3: Social Support Is Informal in Nature

The MHN participants shared that the nature of social support was often informal, typically occurring ad hoc during work conversations and interpersonal connections such as sitting in a car to debrief or going for a drink after work. The MHNs found comfort knowing that someone was always there, and “*just a phone call or text away*” (MHN 5). Only one MHN (MHN 6) referred to social support being a timetabled and structured process, however this related more to clinical supervision. MHN 6 identified a difference between supervision and social support stating *“… definitely supervision is structured and organised. So, it's that sort of calendared and timetabled…there are other times when it would be quite informal and ad hoc*” (MHN 6). MHN 3 stated in their experience “*it's more informal, it it's more kind of the day‐to‐day sort of interpersonal social support that we provide to each other as a as a course of doing what we're doing and caring for each other and supporting each other*”. The informality and ad hoc nature of social support was also raised by MHN 5 who shared “*it's nice after a shift just to have a have a drink together”* or “*sitting in the in my colleague's car, [to say] that was just, that was just a crazy shift*” and, “*It would have been weird to just go home. Does that make sense? It needed to sort of be just talked about. That was really helpful*”.

### 5.4 Theme 4: The Availability of Social Support

The MHN participants reinforced that a feature of social support is that they could access support as frequently as they required. The frequency was determined by the MHNs own needs and could occur as frequently as “*each shift*” (MHN 1 and 4). Social support was also seen to be just a “*phone call away”* (MHN 4) in contrast to more formalised debriefing that lack immediacy. MHN 3 shared “*They're always available. Yeah, I've I never never have to feel that they're too busy or anything like that*”. MHN 4 identified that they often experienced social support by “*Reach[ing] out*” to get “*some sort of balance back*” before going home. It is important to note that all MHN participants in this study had been at their workplace for several years (ranging from 5 to 27 years) and had established professional relationships with their peers.

The integration of online technology as an additional means to provide social support was also expressed. MHN 2 identified “small things…[like] supportive emails” acknowledging that “it doesn't seem much, but it can be a massive help”. Additionally, it was expressed that some MHNs utilise technology to support their well‐being, “Digitally as well, like through different platforms, I think that's, well, that's a good thing or bad thing, I don't know… a lot of them [MHN's] are on a WhatsApp group…That was suggested to us once…but [I] didn't really want want to do that and hear about things when I wasn't at work …I want to keep it quite separate” (MHN 5).

### 5.5 Theme 5: “You Cannot Survive the Industry” Without Social Support

The findings from this research have identified that MHNs highly value social support and consider it as “*integral to our work*” (MHN 6). MHN 2 described that when they feel well supported “*it feels wonderful. You feel safe. You want to go to work. You want to do overtime. You want to do your best. You want to impress them. You want to, you know, go out of your way to make sure that you help the team. And when you don't feel supported, you don't want to do any of those things*”.

The notion of being a member of a team and not being alone was prominent particularly from MHN 5 who reflected that “*no matter how difficult it got, you just knew everyone had each other's back. It was just kind of shared experience. And you're going to get through it together*”. The findings identified that social support is necessary as a protective factor for MHNs to perform their work. This was illustrated by MHN 3, “*I think it's essential, but I think it is actually inherent to what we do. We need to have that because if you don't, then the workplace that you're in becomes incredibly toxic and incredibly difficult to actually function in*”.

In contrast, some participants voiced their disappointment in leadership particularly surrounding a perceived lack of valuing social support. MHN 2 stated that management in their organisation would demonstrate social support by “*showing them the policies, the procedures, you know, the correct way of doing things. Umm, but that's about it. It's never about, you know, do you need any help? Are you doing alright? You know, is there anything we can help you with? Are you coping OK with the environment that you're at the moment for example, I recently heard you got assaulted*”.

Consistent among all the MHN participants was an acute understanding that social support was crucial to being a MHN and functioned as a survival mechanism. The conditions and nature of the work that MHNs do created a need for social support. MHN 4 reflected on advice they frequently provide to nursing students at the start of their career, “*It's something I teach my students is that if you cannot survive the industry, you'll need a therapist*” (MHN 4). Reflections such as this demonstrate both social support and, when needed, professional support (such as seeing a therapist) is recognised as valuable and even necessary resources for MHNs throughout their careers.

## 6 Discussion

The findings of this study underscore the pivotal role of social support in enhancing the professional experiences of MHNs. The emergence of five key themes provides a nuanced understanding of how MHNs perceive, access, and value social support within their work environments. The MHNs who participated in this study brought experience from a range of professional settings, including acute inpatient units—spanning specialty areas such as child and adolescent services—as well as community case management roles. Additionally, several participants were currently working in educational and managerial positions within mental health services, with varying degrees of direct consumer contact. These diverse workplace environments contributed to distinct workplace cultures, which in turn influenced the types and accessibility of social support available to MHNs.

### 6.1 Theme 1: Social Support Is up to You

MHNs in this study emphasised the personal responsibility involved in establishing and maintaining social support networks. This aligns with previous research indicating that nurses often rely on self‐initiated peer connections to cope with workplace challenges. For instance, a qualitative study highlighted that MHNs utilise informal and formal supports to manage workplace risks and stressors, underscoring the importance of self‐driven support mechanisms (Fahy and Moran 2018). The MHN participants in our study resoundingly spoke of social support that takes place informally, most commonly during their shift. The MHNs interviewed highlighted that they primarily found social support among their peers as peer support on the ground which was more accessible than reaching out to formal support services or management. MHN 5 states that the “*chaotic*” nature of the work makes MHNs rely on their peers as a source of social support. This was substantiated by Fahy and Moran (2018) who confirmed that MHNs rely heavily on the informal supports of co‐workers as a social support especially because of the various occupational risks MHNs are exposed to. An example of the positive effect of informal social support sessions was shared by MHN 5 “*Our NUM (Nurse Unit Manager) bought us all … coffee, and we just sat outside and had a pretty informal nurses' business meeting. That was really nice and something different and [we] felt appreciated by the NUM. That was, that was pretty good…*”

### 6.2 Theme 2: Social Support Is About Creating and Relying on Your Allies

The findings from this study underscore the critical importance of immediate peer support among MHNs. Participants emphasised that real‐time assistance from colleagues is more accessible and effective than scheduled, delayed reflections. This immediacy is vital, given the unpredictable and demanding nature of mental health care. While some MHNs acknowledge potential support from senior staff and executives, practical constraints often make peer support the most feasible option. The demanding nature of nursing duties, as described by participants, limits opportunities for scheduled formalised peer support that other allied health professionals receive. Research indicates that spontaneous peer interactions play a significant role in providing social support among nurses. A literature review by Donovan and Greenwell (2021) highlighted that informal, immediate exchanges among nursing peers are crucial for emotional support and stress management. These unplanned interactions foster a sense of camaraderie and resilience, enabling nurses to cope more effectively with the demands of their profession.

The participants in this study resoundingly identified peer support as integral to their coping with workplace demands. Nursing literature supports the perceived importance of peer support for nurses. Watson et al. (2025) claim that peer support has transformative power for nurses with benefits including mitigation of stress, increased sense of belonging and the development of a united professional identity among co‐workers. This also aligns with findings by Jenkins and Elliott (2004) who investigated the impacts of social support on nursing staff in acute mental health settings. In their study, higher levels of social support from colleagues were linked to less emotional exhaustion. Tatsuno et al. (2021) similarly confirmed the protective like factors of social support, recognising the benefits included reduced levels of depression, stress and anxiety.

Interestingly, research has confirmed a correlation between increased social support and increasing age, job title, and years of service among nurses (Shen et al. 2022). The MHNs who participated in this current research ranged from 5 years of experience to 27 years of experience. Evidence suggests that increasing age and experience make it easier to establish interpersonal relationships with colleagues and obtain the individual social support needs of the nurse (Shen et al. 2022). Future research should explore the experiences of MHNs who are new to an organisation, less experienced, or employed on a casual basis, as they may not yet have established strong peer connections. Understanding their perspectives could provide valuable insights into how social support structures can be optimised to benefit all MHNs, regardless of tenure or employment status. The findings from this research have reinforced that for nurses with 5 or more years of experience, the influence of effective social support on MHNs is significant, playing a crucial role in both career longevity and overall well‐being. Strong social support systems are essential for sustaining the nursing workforce, contributing to job satisfaction and professional resilience (Azim and Islam 2018). For MHNs, these benefits are particularly relevant given the emotionally demanding nature of their roles.

### 6.3 Theme 3: Social Support Is Informal in Nature

The informal and spontaneous nature of social support among MHNs, such as debriefing after shifts or casual conversations, plays a significant role in their well‐being. This informality allows for immediate emotional processing and stress relief, which are crucial in high‐pressure environments. This was evidenced by MHN 5 who shared how helpful and supportive “*just to have a drink together*” was after a “*crazy shift*”. A study on nurses' social well‐being found that informal support systems contribute significantly to job satisfaction and motivation, highlighting the value of these spontaneous interactions (Mozaffari et al. 2015). This level of social support was identified as key to processing the shifts events with MHN 5 noting “*it would have been weird to just go home*”. MHNs in this study often preferred receiving social support from colleagues who understand the unique challenges and nuances of their workplace and consumer interactions, rather than relying solely on family or friends at home. While personal support from family and friends may be emotionally beneficial, it often lacks the lived experience and contextual understanding of MHN work that colleagues provide. Workplace social support has been shown to enhance psychological safety, reduce stress, and mitigate burnout among nurses, emphasising its critical role in fostering well‐being and professional resilience (Shahrour et al. 2022).

Literature highlights social support within the workplace—whether emotional, instrumental, or affiliative—can improve job satisfaction, productivity, and coping mechanisms for nurses facing occupational stressors (Hirvikallio et al. 2024; Shahrour et al. 2022). For example, MHNs frequently exposed to workplace violence report reduced stress when supported by colleagues who share similar experiences (Shahrour et al. 2022). This underscores the importance of activating workplace‐based support systems tailored to the specific demands of MHN roles.

However, this form of informal support is often reactive and episodic. While it provides essential emotional reassurance, it does not address the root causes of stress or systemic issues within the workplace. MHN 4 referred to informal support as “*a helpful band‐aid*” acknowledging its limitations in addressing broader challenges. This reliance on informal support also places a burden on individuals to “*proactively establish and maintain*” (MHN 6) their own social support networks, adding to the already high demands of the role.

### 6.4 Theme 4: The Availability of Social Support

The participants appreciated the readily available nature of social support, often accessible as needed during or after shifts. This immediacy is vital in addressing the acute emotional demands of mental health nursing. Research has shown that the perception of available social support is linked to better mental well‐being among nurses, emphasising the importance of having support systems that can be accessed promptly (Ersin et al. 2021).

The use of online technology to facilitate social support was explored in this study. MHNs highlighted the effectiveness of digital communication tools such as emails and WhatsApp messaging platforms. These technologies were seen as valuable channels for exchanging ideas, expressing emotions, providing acknowledgment, and showing gratitude for quality work. While it was clear that technology could serve as an additional means to enhance well‐being among MHNs, caution was advised. As MHN 5 noted, digital platforms may not be everyone's preferred method for receiving social support. This suggests that while technology can be a useful tool, it should be implemented thoughtfully and with consideration for individual preferences in social support mechanisms. Webster et al. (2019) noted that evaluating technological use to support nurses' well‐being had been greatly under‐researched. Developing technology‐based remote social support systems can offer a highly accessible, sustainable, and cost‐effective way to provide support in the future (Webster et al. 2019). Despite limited research in this area, technology‐mediated support was thought to have a mitigating effect on nurses' stress (Xiao et al. 2022). This approach would enable individuals to receive support from anywhere, reducing barriers related to location and resource availability.

The frequency of social support being available “*every shift*” (MHN 1) or “*always available*” (MHN 3) reinforces its necessity as a critical coping mechanism for MHNs, who frequently encounter high‐stress and emotionally charged situations. Given the unpredictable and often volatile nature of their work, MHNs require immediate and consistent access to peer support to process challenging experiences, manage workplace stress, and maintain emotional resilience. Without this level of availability, distressing encounters may accumulate, leading to burnout, emotional exhaustion, and decreased job satisfaction. Studies have shown that frequent access to social support in high‐stress professions, such as nursing, significantly reduces psychological strain and enhances overall well‐being (Ersin et al. 2021). In contrast to formal debriefing sessions, which may be scheduled infrequently, informal, shift‐based social support ensures that MHNs can receive the reassurance and validation they need in real time, fostering a culture of immediate peer‐based intervention.

### 6.5 Theme 5: “You Cannot Survive the Industry” Without Social Support

The consensus among MHNs that social support is essential for professional survival underscores its critical role as a protective factor against workplace stressors. Studies have demonstrated that adequate social support can mitigate the effects of job‐related stress and reduce turnover intentions among nurses, highlighting its importance in sustaining a stable and effective workforce (Yu et al. 2024).

The statement made by one of the participants, “*that you cannot survive the industry*” (MHN 4) without social support, underpins its perceived value, aligning with recent literature including Carmona‐Cobo and Lopez‐Zafra (2022) and Fahy and Moran (2018). Carmona‐Cobo and Lopez‐Zafra (2022) highlight in their study that social support is a vital job resource that directly improves hospital nurses' well‐being and buffers the adverse effects of daily workplace incivility.

MHN 6's statement that social support is “*integral to our work*” highlights its fundamental role in fostering a sense of safety and job satisfaction. Feeling supported leads to increased motivation, with MHN 6 noting, “*you feel safe*” and “*you want to go to work*.” The necessity of social support is further emphasised by MHN 4's assertion that “*if you cannot survive the industry, you'll need a therapist*” underscoring its role in preventing burnout and emotional exhaustion. In Australia, a substantial proportion of nurses experience mental health challenges, with 32.4% reporting depression and 41.2% affected by both anxiety and stress (Maharaj et al. 2019). The effect of mental health challenges is far reaching, extending beyond the nurse's individual well‐being but also negatively impacting their work performance and quality of consumer care (Yu et al. 2024). However, research confirms that strong workplace support networks improve nurse retention, reduce stress, and enhance well‐being (Fahy and Moran 2018), ultimately benefiting both staff and consumer care.

This study highlights the crucial role of social support in the professional lives of MHNs, emphasising the need for both self‐initiated efforts and organisational strategies to create a supportive work environment. Given the demanding nature of mental health nursing and ongoing workforce challenges in Australia, including high rates of burnout and staff shortages, strengthening social support networks is essential for staff retention and well‐being. Establishing formalised support systems alongside existing informal networks could enhance job satisfaction, reduce stress, and improve workforce sustainability. Ultimately, a well‐supported MHN workforce is better equipped to provide high‐quality care, leading to improved outcomes for consumers in mental health services.

## 7 Limitations

This study was contextualised to the specific setting of Victorian mental health services, as well as the time and participant group involved; it therefore does not intend to provide a generalised view of experiences of social support held by MHNs. The study represents the participants responses that were involved in the study and does not intend to generalise all MHNs opinions of social support. As this was a qualitative research project, there is limited transferability of findings to the wider population of MHNs. MHNs who had a strong sense of social support may have elected to participate in the project; however, it appears from the findings that there was a range of levels of social support experienced by the participants. The themes that emerged from this study are important to inform future studies wishing to explore effective social support strategies designed to better support MHNs, in addition to extending to all health professionals working in mental health settings. The findings also go some way to highlighting how workplaces could better support their MHNs.

## 8 Conclusion

This study highlights the critical role of social support in mitigating the challenges faced by MHNs working in high‐stress and emotionally demanding environments. The findings reveal a multifaceted landscape of social support, where informal and formal mechanisms are accessed to varying degrees. Rather than being complementary, these supports intersect in complex ways across the five themes, highlighting individual agency, relational dynamics, and systemic challenges in how MHNs navigate and experience support.

Informal peer support emerged as a cornerstone of workplace well‐being, offering immediate emotional relief, camaraderie, and shared understanding. However, this type of support, while invaluable, is often reactive and places the burden of building and maintaining support networks on individual nurses. Conversely, formal mechanisms, such as clinical supervision and EAPs, provide structured opportunities for professional growth and reflective practice but are frequently underutilised due to barriers such as scheduling conflicts, inconsistent quality, and organisational limitations.

A key takeaway from this research is the need for organisations to strengthen formal support mechanisms while simultaneously fostering a culture that values and enhances informal peer support. This includes proactive interventions, such as regular debriefing sessions, visible and empathetic leadership, and targeted strategies to address systemic stressors like workload management and workplace safety. Furthermore, acknowledging cultural and generational differences in how MHNs perceive and seek social support is essential for creating inclusive and effective support structures.

Ultimately, improving social support for MHNs requires a multifaceted approach that balances the strengths of informal networks with the stability and structure of formal supports. By addressing these needs, healthcare organisations can reduce burnout, enhance job satisfaction, and improve staff retention, creating a more sustainable and supportive environment for those at the forefront of mental health care.

## 9 Relevance for Clinical Practice

Exposing MHNs experiences of social support highlights its critical role in their professional sustainability. Given that support is often informal and reliant on peer relationships, the profession must consider more structured, equitable approaches. This research underscores the need for systemic support mechanisms, as relying solely on peer connections is unsustainable and potentially inequitable. Raising awareness of this issue is essential to safeguarding the well‐being of MHNs and ensuring their retention in clinical roles.

## Disclosure

*Authorship statement*: The authors acknowledge that all authors listed meet the authorship criteria according to the latest guidelines of the International Committee of Medical Journal Editors, and that all authors are in agreement with the manuscript.

## Ethics Statement

The authors have nothing to report.

## Consent

The authors have nothing to report.

## Conflicts of Interest

The authors declare no conflicts of interest.

## Supporting information


**Data S1:** inm70132‐sup‐0001‐DataS1.docx.

## Data Availability

The data that support the findings of this study are available on request from the corresponding author. The data are not publicly available due to privacy or ethical restrictions.
