# Supplementary material for: Silent Strength: The Social Support of Mental Health Nurses
Source: Int J Ment Health Nurs. 2025 Sep 4;34(5):e70132. doi: 10.1111/inm.70132 (PMC12409769; doi:10.1111/inm.70132)
Supplement: Supplementary file 1 — Data S1: inm70132‐sup‐0001‐DataS1.docx. [file INM-34-0-s001.docx]

**COREQ Guidelines:**

**Domain 1: research team and reflexivity**

1. Interviewer/facilitator – Three researchers conducted the interviews; Dr Sinead Barry, Associate Professor Ruby Walter, and Dr Azizur Rahman
2. Credentials – Sinead Barry: PhD, Senior Lecturer, Mental Health Nurse

Ruby Walter: | RN, PhD, BNurs(Hons), GCTE | Acting Assistant Associate Dean Nursing

Azizur Rahman: PhD, Senior Lecturer, Occupational Health and Safety

Katrin Leifels: PhD, Lecturer, Occupational Health and Safety

1. Occupation – Sinead Barry: Senior Lecturer Mental Health Nursing, RMIT University

Ruby Walter: Acting Assistant Associate Dean Nursing, RMIT University

Azizur Rahman: Senior Lecturer, Occupational Health and Safety, RMIT University

1. Katrin Leifels: Lecturer, Occupational Health & Safety, RMIT University
2. Gender – Sinead Barry: Female

Ruby Walter: Female

Azizur Rahman: Male

Katrin Leifels: Female

1. Experience and training – Sinead Barry - senior lecturer in mental health nursing, I have undertaken a PhD in mental health nursing education and been part of and lead research projects leading to publications. I have undertaken all mandatory research and ethics training modules required to undertake research at RMIT University.

Ruby Walter: has over 25 years of experience as a qualitative researcher in nursing. Ruby’s research interests have included a focus on stress management, workforce development, self-concept and graduate nurse experiences. Ruby has completed all mandatory research and ethics training modules required to undertake research at RMIT University.

Azizur Rahman: is a senior lecturer in Occupational Health and Safety (OHS) at RMIT University, Australia. He obtained his PhD in OHS from Swinburne University of Technology (SUT) in Melbourne. Azizur’s research interests have included workplace well-being and equity of working life. Azizur has completed all mandatory research and ethics training modules required to undertake research at RMIT University.

Katrin Leifels: Is currently a lecturer in Occupational Health and Safety. a lecturer in Occupational Health and Safety. Katrin's research activities and her PhD focus on the impact of cultural diversity on the individual wellbeing. Katrin undertook all mandatory research and ethics training modules required to undertake research at RMIT University.

1. Relationship established – No, a relationship was established with participants upon commencement of the interview.
2. Participant knowledge of the interviewer – participants had no prior knowledge of the interviewer until they formally introduced themselves at the commencement of the interview.
3. Interviewer characteristics – Sinead is a mental health nurse so had a genuine interest in hearing perspectives of mental health nurse’s social support. She had never worked at the organisations that the interviewees were employed at, nor had she had previous working relationships with the interviewees

Ruby Walter: Ruby is a registered nurse and an experienced qualitative researcher and interviewer. Ruby was not known to the participants and had not worked at any of the organisations the participants were employed at.

Azizur Rahman: Azizur is a Certified Human Factors Professional which gave him a genuine interest in understanding mental health nurses’ perspectives on social support. He had no prior experience working at the organizations where the interviewees were employed, and he had not previously worked with any of the interviewees either.

**Domain 2: study design**

9. Methodological orientation and theory –A qualitative methodological design was used, as the study aimed to explore the lived experiences and perceived levels of social support among registered nurses working in mental health settings. This approach was chosen to gain a deeper understanding of how social support is experienced and perceived by individuals within this specific professional context. To recruit participants with relevant experiences, purposive sampling was initially employed, followed by snowball sampling to identify additional mental health nurses who met the inclusion criteria and were willing to participate.

10. Sampling – The researchers advertised the information about the research on professional websites and networks. As part of a purposive and snowball recruitment process, responders to the advertisements were contacted.

11. Method of approach – Online advertisement including purposive and snowball sampling from earlier participants

12. Sample size – 6 mental health nurses. This sample size was considered appropriate for an exploratory qualitative study, allowing for in-depth insights while maintaining manageability for detailed thematic analysis.

13. Non-participation – 0

14. Setting of data collection – Interviews were conducted online using MS Teams

15. Presence of non-participants – No

16. Description of sample – Participants had to be a practicing mental health nurses, registered with AHPRA working in Australia.

17. Interview guide – Interview guide was established and approved by University Ethics Committee. They were no pilot interviews conducted.

18. Repeat interviews – No

19. Audio / visual recording – Interviews were audio recorded

20. Field notes – No field notes were made during or after the interview

21. Duration – Interviews lasted for a maximum of 50min

22. Data saturation – In this study, the concept of data saturation was not explicitly discussed or used as a criterion for sample size or data collection closure. The aim of the data collection was to explore the diversity of experiences rather than generalise.

23. Transcripts returned – They were offered, however no participants requested to view them

**Domain 3: analysis and findings**

24. Number of data coders – Two researchers (Sinead Barry and Ruby Walter) coded the data and came together to agree upon the final themes

25. Description of the coding tree – No, we followed Braun and Clarkes approach to thematic analysis.

26. Derivation of themes – No they were derived from the data

27. Software – Data analysis was undertaken manually no software other than MS Word was used

28. Participant checking – They were offered, however no participants requested transcripts

29. Quotations presented – Yes, they were included and identifiable only by participant number

30. Data and findings consistent – Yes

31. Clarity of major themes – Yes

32. Clarity of minor themes – Yes, there has been a range of diverse opinions expressed within the paper. The aim of the research was not to make broad generalisations, rather to showcase diversity and richness in the data.
